# Artificial Hydration at the end of Life in an Oncology Ward in Singapore

**DOI:** 10.4103/0973-1075.73668

**Published:** 2010

**Authors:** Lalit Kumar Radha Krishna, Jissy Vijo Poulose, Cynthia Goh

**Affiliations:** 1 Department of Palliative Medicine, National Cancer Centre, Singapore; 2 Lien Centre for Palliative Care, Duke NUS Graduate Medical School, Singapore

**Keywords:** Artificial hydration, supportive care, survival, terminal cancer

## Abstract

**Aim:**

The objective of this study has been to examine the frequency of use of artificial hydration in terminally ill cancer patients during the last 48 h of life and the occurrence of symptoms specific to hydration status. Other objectives were to find out if artificial hydration has any impact on survival or had any influence on the patterns of use of opioids and sedatives while under palliative care.

**Materials and Methods:**

Retrospective review of case notes of palliative care patients who died in a 95 bedded oncology ward was done. Information on demographic profile, duration of palliative care, medication use and on symptoms related to hydration status was collected. Patients on artificial hydration were compared to those who were not on artificial hydration for the above parameters. Survival curves were plotted for both groups using Kaplan-Meier method.

**Results:**

There were 238 patients of which 55.5% were females. The median age was 62 years and the median duration of palliative care was five days. Artificial hydration was given to 59.2% of patients. There was no significant difference in the incidence of symptoms related to hydration status or in the patterns of medication use between patients who received artificial hydration and those who did not. Kaplan-Meier survival curves did not show any significant survival difference (*P* value=0.9) between the two groups.

**Conclusion:**

Artificial hydration during the last 48 h of life did not have any significant impact on symptoms related to hydration status, medication use or on survival in terminally ill cancer patients under palliative care.

## INTRODUCTION

The provision of food and care to the weak and frail remains a central tenet of many cultures; hence, it is unsurprising that the provision of artificial hydration at the end of life remains a hotly contested area of ethical, cultural and psychosocial debate. Central to this is not just a failure to see out this fundamental humanitarian duty but the fear of hastening death in patients who are allowed to dehydrate in the face of reduced oral intake in the terminal phase. Indeed such a death is often believed to be painful and liable to increase suffering spurring many family members and health professionals to advocate the maintenance of artificial hydration for their loved ones in the terminal phase of a patient’s illness. Other concerns include precipitation of symptoms like delirium, confusion, myoclonus, somnolence, fatigue, neuromuscular irritability, restlessness, thirst, hunger and constipation, particularly in the presence of opioids, benzodiazepines and neuroleptics such as haloperidol.[1–6]

However, there are equally convincing arguments against the implementation of artificial hydration given an increased preponderance to pulmonary edema, peripheral edema, increased respiratory tract secretions, cough and ascites.[3,7] Furthermore, the institution of intravenous hydration therapy can be painful and psychologically distressing, and may result in restricted mobility and a hindrance to family contact.[8]

To our knowledge, no studies on this issue have been conducted in Singapore thus far and the few studies in international medical literature have been equivocal with regard to the merits of artificial hydration and its impact on survival in this stage of life. This dearth of data poses a significant challenge for healthcare professionals confronted with decision making at the end of life.[9] It is this uncertainty that has provided the impetus for this retrospective study.

### Aim

The primary objective of this study has been to ascertain the frequency of use of artificial hydration among the terminally ill cancer patients under palliative care during the last days of life. Secondary objectives were to examine if artificial hydration at the end of life among cancer patients had any impact on survival or on the incidence of symptoms traditionally related to the hydration status. The association between use of artificial hydration and the prescription of opioids, benzodiazepines and haloperidol during the last two days of life was also explored.

## MATERIALS AND METHODS

The case notes of all patients who died in a 95 bedded oncology ward at a tertiary care hospital in Singapore between September 2006 and September 2007 were reviewed. These patients were jointly managed by the oncology and consultative palliative care teams. Patients who were referred to palliative care team less than 24 h before death were not included in the study. A waiver of consent was obtained from the Institutional Review Board. Data on patient demographics, duration of palliative care involvement, use of artificial hydration and the incidence of hydration related symptoms such as respiratory tract secretions, nausea and vomiting, fluid overload, ascites, urinary retention and edema during the last two days of life were collected. Details of use of opioids, benzodiazepines and haloperidol during the last 48 h and 24 h before death were also collected.

Data analysis was carried out using SPSS version 17 including descriptive statistics and survival analysis. Survival was defined as the time between palliative care referral and death and the Kaplan–Meier survival analysis with log rank test was used to compare the survival of patients receiving hydration at the end of life and those who did not. The two groups (artificial hydration *vs* no hydration) were compared for incidence of symptoms related to hydration status. The frequency and dosing regimes of concurrently administered opioids, benzodiazepines and haloperidol during the last two days of life in the two groups were also compared. Opioid doses were converted to oral morphine equivalent (OME) and benzodiazepine doses were converted to parenteral midazolam equivalent (PME) for the purpose of analysis (Appendix). Provision of fluids via intravenous route to hydrate the patient was considered as artificial hydration.

## RESULTS

There were 238 patients in the sample population, of which132 (55.5%) were females. The median age was 62 years (range 15-96 years) and the median duration under palliative care was five days (range 1-113 days). The three most common primary cancers were of lung (17.6%), colon (16.0%) and breast (10.5%). A total of 141(59.2%) patients were given artificial hydration while 40.8% did not receive artificial hydration in their last 48 h of life. The characteristics of patients, primary cancer diagnoses, patterns of use of medications (at the last 24 h) are shown in Table 1.

**Table 1:** Demographic profile, cancer types and medication use

Characteristics	Total number of patients = 238					
	Artificial hydration *N* = 141 (59.2%)			No artificial hydration *N* = 97 (40.8%)		
Age						
Mean	62 years			61 years		
Median	62 (15-96)years			62(22-88)years		
Age group	N		% of Total	N		% of Total
≤30 years	3		1.3	2		0.8
31-60 years	59		24.8	43		18.1
61-90 years	76		31.9	52		21.8
>90 years	3		1.3	0		0
Gender	
Male	69		29.0	37		15.5
Female	72		30.2	60		25.3
Cancer types						
Stomach	12		5	0		0
Colon	20		8.4	18		7.6
Esophagus	2		0.8	1		0.4
Breast	13		5.5	12		5
Lung	25		10.5	17		7.1
Prostate	3		1.3	2		0.8
ENT	8		3.4	5		2.1
Liver	11		4.6	8		3.4
Unknown primary	8		3.4	7		2.9
Other cancers	39		16.4	27		11.3
*Medications (24 h before death)	*N*	% of hydrated patients	*% of Total*	*N*	% of non hydrated patients	*% of Total*
Opioids	111	78.7	46.6	78	80.4	32.7
Benzodiazepines	23	16.3	9.6	13	13.4	5.4
Haloperidol	14	9.9	5.9	13	13.4	5.4

* Some patients were on more than one medication

The spread of age was similar in the two groups. Sixty five percent of the male patients were given artificial hydration while only 54.5% of the female patients were hydrated. (Fisher’s exact test *P* value 0.11) All 12 patients with stomach cancer received artificial hydration compared to only 52% of colon cancer patients.

### Opioids and sedative use

Overall 78.6% of the patients were on opioids, 15.1% were on benzodiazepines and 11.3% on haloperidol for controlling symptoms related to terminal illness. There was no significant difference in the prevalence of use of these drugs between the two groups. Among the hydrated patients 78.7% were on opioids, 16.3% were on benzodiazepines and 9.9% were on haloperidol. For non hydrated patients, similar patterns of use of these medications were observed.(Opioids in 80.4%, benzodiazepines in 13.4% and haloperidol in 13.4% of the non hydrated patients).

Table 2 shows the mean and median doses of opioids, benzodiazepines and haloperidol at 48 h and 24 h before death for the two groups of patients (artificial hydration *vs* no artificial hydration). Among the patients who received opioids during the final 48 h (N=189), the median opioid dose was 60 mg/24 h for patients on artificial hydration and 36 mg/24 h for those not receiving hydration. But this observed difference was not statistically significant (Mann -Whitney U Test, *P* value =0.1). There was also no statistically significant difference with regard to median doses of benzodiazepines or haloperidol between the two groups. The median dose of midazolam was 5 mg/24 h for the hydrated patients while for the non hydrated patients it was slightly higher(7.5 mg/24 h), but this difference was not significant statistically.(Mann -Whitney U Test, *P* value=0.9). For the patients on artificial hydration, the median haloperidol dose was 5 mg/24 h at 24 h before death while for the non hydrated patients it was 3mg/24 h at 24 h before death. This difference was also not statistically significant. (Mann -Whitney U Test, *P* value=0.5). Table 3 shows the dose changes of opioids, benzodiazepines and haloperidol during the last 24 h before death for both groups of patients.

**Table 2:** Doses of opioids and traditional sedatives

Medication	Artificial hydration		No artificial hydration	
Opioid (OME) mg /24 h	At 48 h before death (*N*=107)	*At 24 h before death (*N*=111)	At 48 h before death (*N*=77)	*At 24 h before death (*N*=78)
Mean (95% CI)	113.3 (73.8-152.9)	102.2 (70.7-133.6)	97.8 (56.7-138.9)	96.9 (55.3-138.5)
Median	60	60	36	36
Range	1-1560	1-1440	3-1000	1-1000
BZD^1^ (PME) mg /24 h	At 48 h before death (*N*=18)	†At 24 h before death (*N*=24)	At 48 h before death (*N*=12)	†At 24 h before death (*N*=13)
Mean (95% CI)	6.6 (4.1-9.1)	7.9 (5.1-10.6)	7.8 (4.9-10.6)	7.4 (4.6-10.2)
Median	5	5	6.2	7.5
Range	1-15	1-24	3-15	1-15
Haloperidol (mg)/24 h	At 48 h before death (*N*=15)	‡At 24 h before death (*N*=13)	At 48 h before death (*N*=14)	‡At 24 h before death (*N*=13)
Mean (95% CI)	4.01 (1.7-6.2)	4.6 (2.3-6.9)	4.4 (1.8-7.1)	4.2 (1.3-7.1)
Median	2.75	5	4	3
Range	1-15	1-15	1-19	1-19

* The differences were not statistically significant, (Mann-Whitney U Test, *P* = 0.1);

† The differences were not statistically significant, (Mann-Whitney U Test, *P* = 0.9)

1 Benzodiazepine;

‡ The differences were not statistically significant, (Mann-Whitney U Test, *P* = 0.5)

**Table 3:** Use of opioids and sedatives and dose changes* in the last 24 h

Medication	Artificial hydration		No artificial hydration	
	Number of patients	% of hydrated	Number of patients	% of non hydrated
Opioid				
No opioids	30	21.3	21	21.6
Decrease in dose	29	20.6	24	24.7
No change in dose	46	32.6	34	35.1
<1.5 fold increase in dose	28	19.9	15	15.5
1.5-3 fold increase in dose	4	2.8	1	1
>3 fold increase in dose	4	2.8	2	2
Benzodiazepines			
No benzodiazepines	118	83.7	84	86.6
Decrease in dose	1	0.7	1	1
No change in dose	12	8.5	8	8.2
≤2 fold increase in dose	3	2.1	2	2
>2 fold increase in dose	1	0.7	0	0
Single dose (1 mg-5 mg)	6	4.2	2	2.1
Haloperidol			
No haloperidol	127	90.1	84	86.6
Decrease in dose	1	0.7	2	2.1
No change in dose	10	7.1	9	9.3
<1.5 fold increase in dose	3	2.1	1	1
>2 fold increase in dose	0	0	1	1

* From the previous day’s dose

### Symptoms related to hydration status

The incidence of hydration related symptoms are shown in Table 4. There was no significant difference in the incidence of symptoms related to hydration between the two groups except for symptoms of congestive cardiac failure and edema which were higher in the group with no artificial hydration.

**Table 4:** Specific symptoms related to hydration status

Symptoms	Artificial hydration		No artificial hydration		*P* value*	Exact sig
	N	%	N	%		
Agitation	31	13	22	9.2	*NS*	*NS*
Myoclonus	8	3.4	3	1.3	*NS*	*NS*
Urinary retention	10	4.2	8	3.4	*NS*	*NS*
Constipation	13	5.5	10	4.2	*NS*	*NS*
Confusion	20	8.4	12	5	*NS*	*NS*
Congestive cardiac failure	5	2.1	10	4.2	0.036	0.05
Edema	13	5.5	17	7.1	0.05	0.07
Respiratory depression	0	0	0	0	*NS*	*NS*
Respiratory tract secretions	11	4.6	7	2.9	*NS*	*NS*
Dry mouth	17	7.1	12	5	*NS*	*NS*
Thirst	0	0	0	0	*NS*	*NS*
Hunger	0	0	0	0	*NS*	*NS*
Hiccup	9	3.8	9	3.8	*NS*	*NS*
Loss of consciousness	18	7.6	16	6.7	*NS*	*NS*
Drowsiness	19	8	7	2.9	*NS*	*NS*
Nausea and vomiting	14	5.9	15	6.3	*NS*	*NS*
Insomnia	15	6.3	10	4.2	*NS*	*NS*
Ascites	13	5.5	11	4.6	*NS*	*NS*
Itching	1	0.4	0	0	*NS*	*NS*
Sudden collapse	0	0	1	0.4	*NS*	*NS*

* NS = *P* value > 0.05

### Survival

**Figure 1:**
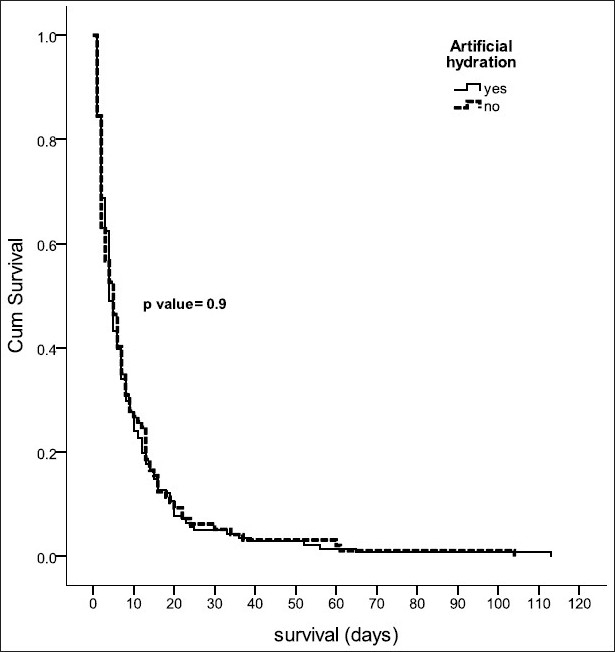
Survival analysis

Kaplan-Meier Survival analysis did not reveal any significant difference in survival (from palliative care referral to death), between the two groups of patients. (Log rank test, *P* value=0.9 as shown in Figure 1.

## DISCUSSION

Our study has shown that a good proportion (59.2%) of the patients in our oncology ward received artificial hydration during the last 48 h before death. This is similar to the reports from other Asian countries like Taiwan and Korea where the proportion of cancer patients receiving artificial hydration during the last 48 h before death was 53.1% and 75% respectively.[10,11]

Our findings suggest that artificial hydration at the very end of life in terminally ill cancer patients does not provide any survival benefit. We also did not observe any significant difference in the occurrence of hydration-related symptoms between the hydrated patients and the non hydrated patients. In fact, the patients who were not on artificial hydration were found to have higher incidence of congestive cardiac failure and edema. However, our data does not allow too much to be drawn from this, given that the indications for the provision and cessation of hydration werenot collected.

An interesting finding that we observed was the apparent reduction in the mean and median doses of opioids in the non hydrated group of patients although the difference was not statistically significant. Indeed dehydration has been postulated to provide an endogenous anesthetic effect through a combination of the resultant ketoacidosis from a reduced caloric intake and the accumulation of endorphins. Therefore, the doses of opioids may show a decreasing trend among those who are dehydrated.[12] We did not observe any significant difference in the doses of benzodiazepines and haloperidol even though it was postulated by some authors that dehydration causes accumulation of these medications and therefore a decrease in the dose of these medications has to be expected among the dehydrated patients.[13] The frequency of use and dose changes of opioids and these sedatives were similar in both groups.

The advantages and disadvantages of artificial hydration in dying patients have been listed by several authors.[3,14] Among the putative advantages of not providing artificial hydration is a reduced urine output, which in turn is associated with a reduced incidence of incontinence and subsequent catheterization. Also stated is an associated reduction of gastrointestinal secretions, which in turn reduces the incidence of vomiting and its sequelae. Evidence also points to a decrease in respiratory tract secretions, cough and the need for suction.[14,15] Cessation of routine hydration of all patients in the terminal phase has been shown to have decreased the incidence of peripheral edema, ascites and cerebral edema in terminally ill patients.[8] Conversely the advantages of providing artificial hydration include prevention of precipitation of symptoms of delirium, myoclonus, confusion and agitation as reported by some authors.[1,15]

Absence of significant differences in the incidence of hydration-related symptoms among our patients supports Waller *et al*’s,[16] findings that hydration does not provide any clinical benefit in the terminal phase. Yet this may be due in part to the very nature of a retrospective study where the data was collected post event. Moreover, data on the duration of provision of hydration for these patients was not collected and given the short duration of study we have not looked into the biochemical profile of our patients to determine whether the patients had any clinically significant electrolyte imbalance. The decision on provision of hydration might have been made based on the preexisting symptoms. It is postulated that the main reason for cessation of hydration in those patients not artificially hydrated at the end of life was primarily driven by the presence of congestive cardiac failure and edema and the consequent decision to avoid compounding of these symptoms. These patients mostly would have been effectively ‘pre-hydrated’ and thus unlikely to develop symptoms of dehydration so rapidly. This, however, does not take into account the effects of ‘third spacing’. Again there are other confounders such as the effect of low albumin and possibly increased vascular permeability which may have prevented these patients utilizing their ‘stores’ of excess hydration. Further study is certainly warranted.

## CONCLUSION

Decision making on providing artificial hydration to terminally ill patients is a challenge for health care professionals. There is no conclusive evidence in the literature to make any recommendations with regard to provision of medically assisted hydration. The stated advantages and disadvantages remain largely anecdotal. Therefore any decision making at the end of life needs to be sensitive to the wishes, goals and concerns of the patients and family with the proviso that such an action is not injurious to the patient. Education on the reduced need for nutrition and hydration in this phase is imperative and needs to be actively propagated. Similarly health professionals need to engage patients in discussions regarding their preferences at the end of life and the options available to them. Clarity with regard to their preferred place of care will also aid in the decision making process.

While more detailed study is required on the ills and benefits of artificial hydration, it is hoped that this paper will have an effect of inciting considered reflection of all facets of patients’ care before commencing artificial hydration.

## Appendix

**Appendix:** Opioid conversion

Route	Types of opioids and doses	OME mg
Oral	Morphine 1 mg	1
Parenteral	Morphine 1 mg	3
Transdermal	Fentanyl 12 mcg	30
Parenteral	Fentanyl 12 mcg	30
Oral	Tramadol 5 mg	1
Oral	Oxycodone 1 mg	2
Oral	Codeine 10 mg	1

Benzodiazepine conversion; Diazepam 10 mg = Clonazepam 0.5 mg = Lorazepam 1 mg = Parenteral Midazolam 5mg; OME = Oral morphine equivalent
